# Bayesian estimation for the random moderation model: effect size, coverage, power of test, and type І error

**DOI:** 10.3389/fpsyg.2023.1048842

**Published:** 2023-07-03

**Authors:** Dan Wei, Peida Zhan

**Affiliations:** ^1^Faculty of Psychology, Beijing Normal University, Beijing, China; ^2^Shenzhen Bao’an Institute of Education Sciences, Shenzhen, China; ^3^School of Psychology, Zhejiang Normal University, Jinhua, China

**Keywords:** random moderation model, heteroscedasticity, Bayesian estimation, moderation analysis, two-level regression model

## Abstract

The random moderation model (RMM) was developed based on a two-level regression model to cope with heteroscedasticity in moderation analysis, and normal-distributed-based maximum likelihood (NML) estimation was developed to estimate the RMM. To present an alternative to the NML, this article discusses the effectiveness of Bayesian estimation for the RMM, aiming to explore a more practical method using the popular software Mplus. Through a simulation study, the RMM based on Bayesian estimation was investigated and compared to maximum likelihood (ML) estimations, including the NML and the default ML estimation in Mplus. The results indicated that the Bayesian approach outperformed the two ML estimations. It showed (a) higher accuracy for estimation of the effect size of the moderation effect; (b) higher 95% credibility interval coverage of the true value of the moderation effect; and (c) well-controlled and more stable type I error rates, while powers comparable to the ML estimations were provided.

## Introduction

A *moderator* is defined as a variable *Z* that affects the direction or strength of the relationship between independent variable *X* and dependent variable *Y* (e.g., MacKinnon, 2008; Hayes, 2013). The moderation effect is traditionally evaluated by including product term *XZ* in the regression model of *Y* on *X*, which is called the moderated multiple regression model (MMRM) (e.g., Aiken and West, 1991). According to the estimate results of the MMRM, if the regression coefficient of *XZ* is statistically significant in terms of the *p*-value of the *F-* or *t*-tests, the moderation effect is approved (e.g., Laird and Ware, 1982; Demidenko, 2004). This statistical significance test relies on the assumption of homoscedasticity across different values of *Z* or *X* (Aguinis et al., 1999; Maronna et al., 2006). A violation of this assumption possibly results in an inflated type I error rate, as well as an inflated type II error rate, which could further exacerbate the low power of the moderation test (Alexander and DeShon, 1994; Aguinis and Pierce, 1998). However, it is difficult for this assumption to be true in practice because it is vulnerable to being affected by longer-than-normal tails, outliers, or unidentical populations (Aguinis et al., 1999; Yuan et al., 2014). It is necessary to treat such samples as stemming from heterogeneous populations (Marcoulides and Trichera, 2019).

As an extension of the MMRM, Yuan et al. (2014) argued for a two-level regression model to conduct the moderation analysis, denoted as random moderation model (RMM) in this article. Depending on the two-level structure, the RMM can cope with the problem of heterogeneous residuals and answer the question of how much moderator variable *Z* explains the effect of *X* on *Y*. The RMM can be estimated using the normal-distribution-based maximum likelihood (NML), which can be conducted using a private R package, *NML.R*, developed by Yuan et al. (2014).^1^ The NML should have properties similar to the maximum likelihood (ML) because both of them approximate estimations based on integral calculations of the likelihood function (Le Cam, 1990; Millar, 2011). However, utilization of ML in the two-level context has been indicated to be more difficult because the dimensions of numerical integration can increase rapidly (Marsh et al., 2004; Asparouhov and Muthén, 2021). Therefore, further research is needed to discuss the efficiency of the NML in the application of the RMM in more detail.

The moderation effect in the RMM using the NML estimation is approved through a significance test for the fixed slope in the level-two model, which corresponds to the coefficient of *XZ* in the MMRM. As methodologists make a growing number of warnings about overreliance on the *p*-value (Bakan, 1966; American Statistical Association, 2016), alternative methods have become increasingly popular to avoid making inferences directly according to the significance test based on the null hypothesis. On one hand, various effect sizes, such as *R*-square, have been investigated to quantitatively report and interpret inferential results (Kelley and Preacher, 2012; Cumming, 2014) instead of simply categorizing the statistical results as *Yes* or *No* (e.g., Yuan and MacKinnon, 2009; Lee, 2016). On the other hand, Bayesian estimation provides a competitive alternative to traditional ML estimation because it can provide a more comprehensive interpretation of the results in terms of not only the statistical significance but also the probability that a given parameter is above a cut-off value or within a given interval (e.g., Yuan and MacKinnon, 2009; Lee, 2016). For example, the moderation effect in the Bayesian approach can be approved when the 95% credibility interval of the coefficient of *XZ* does not contain zero, which indicates that there is a 95% probability that the true value of the regression coefficient lies within the interval limits without zero. In addition, the Bayesian approach has the advantage of being flexible and feasible for use with various complex models, rendering it easily generalized in application. By contrast, NML estimation is less friendly to empirical researchers, as the private package *NML.R* is not officially certificated and, thus, a tutorial manual is not available. Further, application of NML estimation based on *NML.R* is limited because the given estimation process in the source code, such as the convergence criterion, is difficult to modify for different situations. Therefore, the present article aims to explore a more practical method for estimation of the RMM that can be implemented in much more common software—Mplus (Muthén and Muthén, 1998–2012). In this study, utilization of Bayesian estimation in RMM is investigated and compared to NML estimation using *NML.R* package (denoted as NML.R). In addition, given that NML.R is implemented through the private R package, to ensure the accuracy of the study results, we additionally used the default ML estimation with robust standard errors via Mplus (denoted as MLR.M).^2^ The NML.R is the ML estimation based on the normal distribution with conventional standard errors, which assumes the data are normal distributed (Yuan et al., 2014). By contrast, the MLR.M is the ML estimation with standard errors and a chi-square test statistic that are robust to non-normality and non-independence of observation. For normally distributed data, these two estimations would have the same estimation results theoretically.

### MMRM and RMM

The typical MMRM of the regression of *Y* on *X* with moderator variable *Z* can be written as


(1) $$y_{i}=\beta_{0}+\beta_{1}x_{i}+\beta_{2}z_{i}+\beta_{3}x_{i}z_{i}+e_{i},$$

where the product term *XZ* is also called the interaction term, and the moderation effect is quantified as the coefficient $\beta_{3}$. $\beta_{1}$ and $\beta_{2}$ describe the magnitude of the main effects of *X* and *Z* on *Y,* respectively. The error term $e_{i}$ is assumed to be normally distributed with homogenous variance. Based on the MMRM, Yuan et al. (2014) allowed these regression coefficients to vary across individuals, where the variation can be partly explained by the moderator variable. The RMM corresponding to model (1) can be written as a two-level regression model,


(2a) $$\mathrm{Level}\;1:y_{i}=\beta_{i0}+\beta_{i1}x_{i}+e_{iy},$$

(2b) $$\mathrm{Level}\;2:\beta_{i0}=\gamma_{00}+\gamma_{01}z_{i}+\varepsilon_{i0},$$

(2c) $$\beta_{i1}=\gamma_{10}+\gamma_{11}z_{i}+\varepsilon_{i1},$$

where $\beta_{i0}$ and $\beta_{i1}$ vary as individuals. $\gamma_{00}$, $\gamma_{01}$, $\gamma_{10}$, and $\gamma_{11}$ are constant coefficients in the level-two model. $e_{iy}$,$\;\varepsilon_{i0}$, and $\;\varepsilon_{i1}$ are assumed to be normally distributed as *N*(0,$\;\delta_{e_{iy}}^{2}$), *N*(0,$\;\delta_{\varepsilon_{i0}}^{2}$), and *N*(0,$\;\delta_{\varepsilon_{i1}}^{2}$), respectively, and $e_{iy}$ is independent of ($\varepsilon_{i0}$, $\;\varepsilon_{i1}$). Essentially, the RMM can be viewed as a special case of the multilevel model (Snijders and Bosker, 2011). According to Yuan et al. (2014), the RMM can be estimated using NML estimation, which is based on the normal distribution assumption.

To compare the RMM with the MMRM, replace $\beta_{i0}\;$and $\beta_{i1}$ in Eq. 2a with Eqs 2b, 2c; then, the RMM can be written as


(3) $$y_{i}=\gamma_{00}+\gamma_{10}x_{i}+\gamma_{01}z_{i}+\gamma_{11}x_{i}z_{i}+e_{iy}+\varepsilon_{i0}+x_{i}\varepsilon_{i1},$$

The residuals, consisting of $e_{iy}+\varepsilon_{i0}+x_{i}\varepsilon_{i1}$, vary across different groups or individuals characterized by *X*. It should be noted that Eq. 1 is a special case of Eq. 3 when $\delta_{\varepsilon_{i1}}^{2}$ equals zero. The coefficients $\gamma_{00}$, $\gamma_{10}$, $\gamma_{01}$, and $\gamma_{11}$ in Eq. 3 are equivalent to $\beta_{0}$, $\beta_{1}$, $\beta_{2}$, and $\beta_{3}$, respectively, in Eq. 1.

The RMM can answer the question of how much *Z* moderates the effect of *X* on *Y*. It can be assessed by effect size *R*-square, which is calculated as the percentage of the variation of random coefficient $\beta_{i1}$ explained by *Z*,


(4) $$R^{2}=\frac{\gamma_{11}^{2}\sigma_{Z}^{2}}{\gamma_{11}^{2}\sigma_{Z}^{2}+\delta_{\varepsilon_{i1}}^{2}},$$

where $\sigma_{Z}^{2}$ is the variance of the observed variable *Z*. In contrast to the RMM, the MMRM considers that the between-person variation of $\beta_{i1}$ can be completely explained by the moderator variable *Z*, ignoring the possible random errors of the moderation effect that can cause heteroscedasticity. Therefore, the value of *R*-square defined in Eq. 4 would always be equal to 1 in the MMRM. This ignores violation of the assumption of homoscedasticity and normality and may reduce the accuracy of hypothesis testing in moderation effect estimation (Alexander and DeShon, 1994; Aguinis and Pierce, 1998).

The homoscedasticity assumption of the MMRM implies that the individuals are sampled from one population; hence, the random error is normally distributed with a constant variance. However, a review of the literature by Aguinis et al. (1999) found that around 50% of studies using MMRM violated this assumption. By contrast, in RMM, the variance of the random error can be varied across different individual observations; the error term, $e_{iy}+\varepsilon_{i0}+x_{i}\varepsilon_{i1}$, describes the heterogeneous residuals, which vary across different individuals characterized by *X*. The variance of random error is $\mathrm{var}\left(e_{iy}+\varepsilon_{i0}+x_{i}\varepsilon_{i1}\right)=\delta_{e_{iy}}^{2}+\delta_{\varepsilon_{i0}}^{2}+2x_{i}\mathrm{cov}\left(\varepsilon_{i0},\varepsilon_{i1}\right)+x_{i}^{2}\delta_{\varepsilon_{i1}}^{2}$; hence, the heteroscedasticity increased non-linearly with *X*.

### Bayesian parameter estimation

In Bayesian estimation, we combine beliefs about the parameters with evidence from an observed set of data to draw conclusions about unknown model parameters. A Bayesian model consists of two components: a prior distribution that specifies assumptions about the unknown parameters independent of the data, and a statistical model that defines the distribution of data—often a likelihood function. The Bayesian approach seeks the posterior distribution of the model parameter, which encompasses the complete topography of peaks, valleys, and plateaus, as opposed to the frequentist approach, which seeks a point estimate of each model parameter. The likelihood of the data given the parameters and the prior distribution of the parameters are multiplied to obtain the posterior distribution of parameters given the data. The posterior mean (or median, or mode) serves as a summary of central tendency, whereas the posterior standard deviation serves as a description of variability when describing the posterior distribution. Complex posterior distributions, however, may be exceedingly difficult to manage in practical computing. The posterior distribution of Bayesian models’ means and standard deviations may be estimated via MCMC simulations. A large sample of representative random values from a posterior distribution should be used in order to evaluate its attributes. A simulation known as a Monte Carlo process samples a large number of random variables from an interesting posterior distribution. For further information on the Bayesian MCMC approach, readers might see Kruschke (2015) and Levy and Mislevy (2016).

Bayesian estimation has been gaining in popularity (Kruschke, 2015; Van de Schoot et al., 2017), as it provides a flexible tool to fit various complex models, such as heterogeneous or nonnormal models (Rast et al., 2012; Oravecz and Muth, 2018), which are difficult or inefficient to estimate using regular ML estimation (e.g., Wang and McArdle, 2008; Chow et al., 2011; Song et al., 2011; Muthen and Asparouhov, 2012). For example, Zhang et al. (2013) derived robust growth curve models using Student’s *t* distribution and used the Bayesian approach to estimate the model. Rast et al. (2012) applied the Bayesian approach to the estimation of a mixed-effects location scale model (Hedeker et al., 2008, 2009), which allowed explanations of both between-person and within-person variations in a growth trajectory using explanatory variables in longitudinal analysis (e.g., Hoffman, 2007). Wang and Preacher (2015) indicated that their Bayesian approach yielded unbiased estimates and higher or comparable power to the ML estimation in moderated mediation analysis (e.g., Hayes, 2013; Hayes and Preacher, 2013).

More recently, the Bayesian approach was directly applied in the estimation of a two-level moderated mediation model (Liu et al., 2022), which was further developed based on the RMM to cope with situations where the strength of an indirect (mediation) effect depends on the moderator variable (e.g., Preacher et al., 2007). Liu et al. (2022) focused on the moderated mediation effect, while this study focuses on the moderation effect. In addition, the estimation of the Bayesian approach remains to be investigated because evidence is rare for the performance of Bayesian estimation in two-level models with an actual non-clustered data structure. In this study, Bayesian estimation for the RMM was explored to discuss the efficiency of different estimation methods. The Bayesian estimation was implemented using Mplus software under default settings in order to increase the practicality and operability of this approach. The code used for the estimation in Mplus is given in the appendix of this article for reference to interested empirical researchers.

## Stimulation study

### Data generation

A simulation study was conducted to explore the performance of the RMM based on Bayesian estimation via Mplus. Referring to previous simulations in relevant fields, four factors were manipulated at several representative levels interactively (e.g., Wang and Preacher, 2015; Liu et al., 2022), including (a) sample size, *N* = 100 and 500; (b) magnitude of the error variance, $\delta_{\varepsilon_{i1}}^{2}$= 0, 0.50, and 1; (c) magnitude of the regression coefficients, γ = 0.29 and 0.59; and (d) correlation coefficient between *X* and *Z*, ρ = 0 and 0.50.

The observed independent variable *X* and moderator variable *Z* were generated from the bivariate normal distribution *N*(μ,Σ) with zero means and unit variances, and the correlation coefficients between the two variables are set as ρ. The error term was simulated as the combination of three separate residual errors $e_{iy}+\varepsilon_{i0}+x_{i}\varepsilon_{i1}$ that were uncorrelated with each other and with *X* and *Z.* The $e_{iy}$ and $\varepsilon_{i0}$ were extracted from standard normal distribution *N*(0,1), and $\varepsilon_{i1}$ was generated from normal distributions with consistent zero means and different values of variance $\delta_{\varepsilon_{i1}}^{2}$. The regression coefficients $\gamma_{00}$, $\gamma_{10}$, $\gamma_{01}$, and $\gamma_{11}$ were all fixed equal and set as γ, discussing the power for evaluation of the moderation effect. Then, based on the above 24 $\left(2\left(N\right)\times 3\left(\delta_{\varepsilon_{i1}}^{2}\right)\times 2\left(\rho \right)\times 2\left(\gamma \right)\right)$ conditions, another 24 simulations that set $\gamma_{11}$ as zero were correspondingly investigated, where the type I error rate of the moderation effect estimation was discussed. The dependent variable *Y* was generated according to Eq. 3. Finally, a total of 48 conditions were conducted, with 500 replications under each of these conditions.

### Reparametrization of RMM

**Figure 1:**
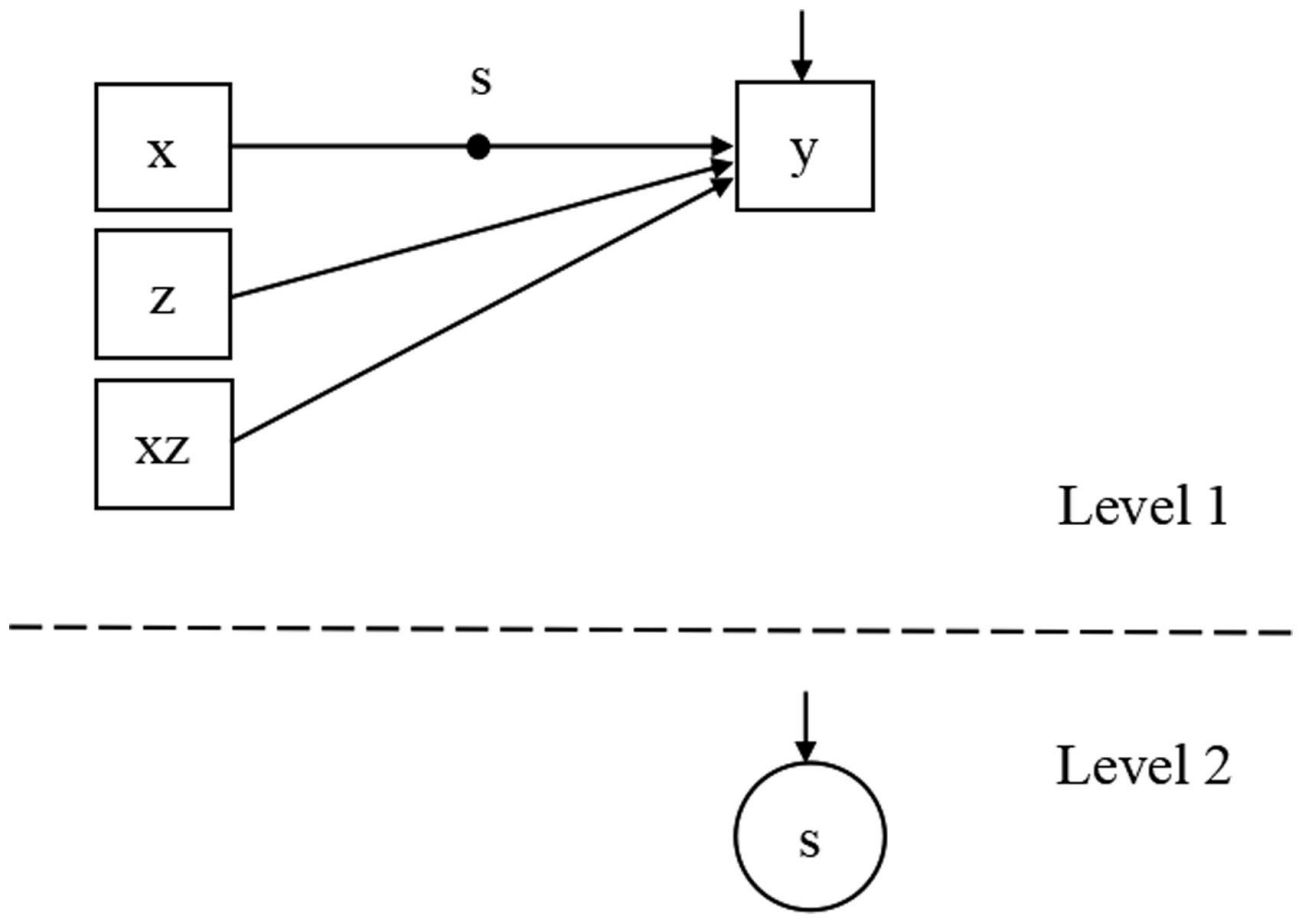
The path diagram of the RMM estimated in the simulation study.

To estimate the RMM in Mplus with the essentially single-level data, a cluster variable has been added to the data set. This variable consisted of a sequence of numbers without repeated values, indicating that each group had only one individual, which was used to “trick” the software into thinking that the data have two levels (Liu et al., 2022). Considering that both $e_{iy}\;\mathrm{and}\;\varepsilon_{i0}$ represent homogenous residuals according to Eq. 3, the estimation of the combined error variance (denoted as $\delta_{\varepsilon_{iy}}^{2}$) is expected to perform better than the estimates of the two ($\delta_{e_{iy}}^{2}$ and $\delta_{\varepsilon_{i0}}^{2}$) separately (Yuan et al., 2014). Therefore, in Mplus, the RMM can be reparametrized as


(5a) $$\mathrm{Level}\;1:y_{i}=\gamma_{00}+s_{i}x_{i}+\gamma_{01}z_{i}+\gamma_{11}x_{i}z_{i}+\varepsilon_{iy},$$

(5b) $$\mathrm{Level}\;2:s_{i}=\gamma_{10}+\varepsilon_{i1},$$

where $\varepsilon_{iy}=e_{iy}+\varepsilon_{i0}$. And the corresponding path diagram is given in Figure 1. If estimating the RMM according to Eqs 2a–2c directly, an extra error term needs to be estimated under a redundant restriction that $e_{iy}$ and $\varepsilon_{i0}$ are independent. This should reduce the estimation accuracy compared to Eqs 5a, 5b.

### Analysis

In Mplus, default (noninformative) prior and convergence criterion were applied in the Bayesian estimation, which was denoted as Bs.M. The default priors were widely applicable to various models and data sets (Congdon, 2001). The default Gelman–Rubin convergence criterion determined convergence by assessing within- and between-chain variability for each parameter based on the potential scale reduction (PSR) factor (Gelman et al., 2003), and convergence was considered achieved if the PSR statistic was less than 1.05 (Brooks and Gelman, 1998).

For the Bs.M, three Markov chains were drawn for each estimation, and the replication would stop for each chain when the convergence criterion was reached or the given maximum of 10,000 replications was produced. The code used to specify the Bs.M was shown in Appendix A. In addition, the ML estimation was discussed for a comparative perspective, including the NML.R and the MLR.M. The code used to conduct the MLR.M was shown in Appendix B. For additional details of the implementation of *NML.R* package, please see Yuan et al. (2014).

We explored estimation of the above three methods for estimation of the moderation effect, in terms of the accuracy of regression coefficients and effect size *R*-square. The estimation accuracy was evaluated through average of |bias| and mean square error (MSE). Let α and $\hat{\alpha }$ denote the true and estimated values, respectively, of a parameter; then, the average of |bias| and MSE could be calculated by.


(6) $$Averageof\left|bias\right|=\frac{\sum_{r=1}^{500}\left|{\hat{\alpha }}_{r}-\alpha_{r}\right|\;}{500},$$

(7) $$MSE=\frac{\sum_{r=1}^{500}{\left({\hat{\alpha }}_{r}-\alpha_{r}\right)}^{2}\;}{500}.$$

The coverage, the power of test, and type I error were further computed based on the 95% credibility interval for the Bs.M and the 95% confidence interval for two ML estimations (for simplicity, the 95% credibility interval and 95% confidence interval are both denoted as 95% CI). Specifically, the coverage was calculated as the proportion of the 95% CI of ${\hat{\gamma }}_{11}$ contained the true value of $\gamma_{11}$ in 500 replications; The power of test was calculated as the proportion of 95% CI of ${\hat{\gamma }}_{11}$ did not contained zero in 500 replications when $\gamma_{11}\neq 0$; The type I error was calculated as the proportion of 95% CI of ${\hat{\gamma }}_{11}$ did not contained zero in 500 replications when $\gamma_{11}=0$.

## Results

### Estimation accuracy of coefficients

For the estimation of ${\hat{\gamma }}_{00}$, ${\hat{\gamma }}_{01}$, and ${\hat{\gamma }}_{10}$, the three methods, Bs.M, MLR.M, and NML.R, had similar performance and very small MSEs that approached zero in most conditions (ranging from 0.000 to 0.003 when *N* = 500 and from 0.000 to 0.016 when *N* = 100). Tables 1 and 2 showed the accuracy of ${\hat{\gamma }}_{11}$ under the conditions that $\gamma_{11}$ ≠ 0 and $\gamma_{11}$ = 0, respectively. In addition, the accuracy of error variances were given in Appendix C for interested researchers (see Tables I–IV).

**Table 1:** Averages of |bias| and MSEs of ${\hat{\gamma }}_{11}$ under conditions that discuss the power ($\gamma_{11}$ ≠ 0).

	$\delta_{\varepsilon_{i1}}^{2}$	Cor	Averages of |bias|			MSEs		
			Bs.M	MLR.M	NML.R	Bs.M	MLR.M	NML.R
γ = 0.29 (*N* = 500)	1	0	0.063	0.062	0.063	0.006	0.006	0.006
	1	0.50	0.062	0.062	0.062	0.006	0.006	0.006
	0.50	0	0.059	0.059	0.059	0.005	0.005	0.006
	0.50	0.50	0.058	0.058	0.058	0.005	0.005	0.005
	0	0	0.052	0.052	0.057	0.004	0.004	0.005
	0	0.50	0.046	0.046	0.046	0.003	0.003	0.003
γ = 0.29 (*N* = 100)	1	0	0.155	0.150	0.154	0.037	0.035	0.037
	1	0.50	0.138	0.137	0.137	0.030	0.030	0.030
	0.50	0	0.139	0.135	0.137	0.029	0.029	0.030
	0.50	0.50	0.128	0.128	0.132	0.026	0.026	0.028
	0	0	0.119	0.116	0.113	0.022	0.022	0.022
	0	0.50	0.109	0.104	0.110	0.019	0.017	0.019
γ = 0.59 (*N* = 500)	1	0	0.065	0.064	0.064	0.007	0.006	0.006
	1	0.50	0.063	0.062	0.062	0.006	0.006	0.006
	0.50	0	0.061	0.060	0.060	0.006	0.006	0.006
	0.50	0.50	0.054	0.054	0.054	0.005	0.005	0.005
	0	0	0.053	0.053	0.050	0.004	0.004	0.004
	0	0.50	0.045	0.044	0.044	0.003	0.003	0.003
γ = 0.59 (*N* = 100)	1	0	0.150	0.147	0.147	0.035	0.034	0.034
	1	0.50	0.146	0.144	0.147	0.033	0.032	0.034
	0.50	0	0.141	0.134	0.141	0.032	0.029	0.032
	0.50	0.50	0.128	0.127	0.131	0.025	0.025	0.026
	0	0	0.124	0.118	0.126	0.024	0.022	0.026
	0	0.50	0.116	0.111	0.111	0.021	0.019	0.020

γ represents the true value of fixed coefficients $\gamma_{00},\gamma_{01},\gamma_{10},$ and $\gamma_{11}$. Cor represents the true value of the correlation coefficient between variables *X* and *Y*.

**Table 2:** Averages of |bias| and MSEs of ${\hat{\gamma }}_{11}$ under conditions that discuss type I error ($\gamma_{11}$ = 0).

	$\delta_{\varepsilon_{i1}}^{2}$	Cor	Averages of |bias|			MSEs		
			Bs.M	MLR.M	NML.R	Bs.M	MLR.M	NML.R
γ = 0.29 (*N* = 500)	1	0	0.062	0.062	0.063	0.006	0.006	0.006
	1	0.50	0.057	0.057	0.057	0.005	0.005	0.005
	0.50	0	0.059	0.058	0.058	0.005	0.005	0.005
	0.50	0.50	0.054	0.054	0.054	0.005	0.005	0.005
	0	0	0.049	0.049	0.051	0.004	0.004	0.004
	0	0.50	0.048	0.047	0.045	0.004	0.004	0.003
γ = 0.29 (*N* = 100)	1	0	0.143	0.136	0.139	0.032	0.031	0.032
	1	0.50	0.141	0.135	0.140	0.031	0.030	0.032
	0.50	0	0.149	0.146	0.149	0.034	0.033	0.034
	0.50	0.50	0.132	0.128	0.132	0.027	0.025	0.026
	0	0	0.132	0.125	0.130	0.026	0.024	0.026
	0	0.50	0.117	0.115	0.123	0.022	0.020	0.023
γ = 0.59 (*N* = 500)	1	0	0.063	0.064	0.064	0.006	0.006	0.006
	1	0.50	0.056	0.057	0.057	0.005	0.005	0.005
	0.50	0	0.062	0.062	0.062	0.006	0.006	0.006
	0.50	0.50	0.053	0.052	0.052	0.004	0.004	0.004
	0	0	0.053	0.053	0.057	0.004	0.004	0.005
	0	0.50	0.046	0.046	0.047	0.003	0.003	0.003
γ = 0.59 (*N* = 100)	1	0	0.158	0.155	0.158	0.038	0.038	0.040
	1	0.50	0.144	0.141	0.142	0.033	0.032	0.032
	0.50	0	0.146	0.142	0.145	0.031	0.030	0.032
	0.50	0.50	0.126	0.125	0.128	0.025	0.025	0.026
	0	0	0.118	0.115	0.124	0.021	0.020	0.023
	0	0.50	0.109	0.106	0.111	0.020	0.019	0.021

γ represents the true value of fixed coefficients $\gamma_{00},\gamma_{01},\gamma_{10},$ and $\gamma_{11}$. Cor represents the true value of the correlation coefficient between variables *X* and *Y*.

Results indicated that the accuracy of the estimations of the coefficients was hardly influenced by the magnitude of their true values, as the MSEs under the condition γ = 0.29 were similar to those under the condition γ = 0.59, whether the true value of $\gamma_{11}$ was the same as the other coefficients (see Table 1) or was zero (see Table 2). The three methods performed better and more similarly to each other under a larger than a small sample size. As the true value of $\delta_{\varepsilon_{i1}}^{2}$ increased, the three methods showed decreases in accuracy. The influence from different true values of the correlation coefficient between *X* and *Z* was very small and could be ignored. In sum, the Bs.M, MLR.M, and NML.R showed little difference for the estimation of coefficient parameters according to MSEs, especially when the sample size was large.

However, the NML.R was prone to nonconvergent results. As *NML.R* package reported, the convergence rates of the NML.R under conditions with a small sample size (ranging from 57.2 to 97.6%) were lower than those under conditions with a large sample size (ranging from 90.6 to 100%). While the Bs.M had a few nonconvergent results only under the condition *N* = 100, the convergence rates of the Bs.M (ranging from 97.6 to 99.4% when *N* = 100) were much higher than those of the NML.R. Therefore, the present and subsequent results of the NML.R and Bs.M were calculated based on the convergent samples under the conditions with nonconvergent data sets. What’s more, it was found that the NML.R might provide a negative value for the estimation of ${\hat{\delta }}_{\varepsilon_{i1}}^{2}$ in most cases (more than 60% in all conditons) over 500 replications. Those cases with a negative ${\hat{\delta }}_{\varepsilon_{i1}}^{2}$ have also been removed from the summarized table of the NML.R. The MLR.M could achieve convergence under all the conditions.

### Coverage, power of test, and type І error of the moderation effect

The powers and type І error rates in all the conditions were presented in Table 3. According to the power, the three methods performed much better when the sample size and true value of γ were larger (*N* = 500, γ = 0.59) than in the other conditions. The MLR.M and NML.R had very similar results in power, while the Bs.M showed slightly lower values under almost all the conditions and had no advantage over the MLR.M or NML.R. Nevertheless, the three methods showed little difference in terms of the criterion that good power should be larger than 0.80, except for 2 of 24 conditions (where *N* = 100, γ = 0.59, and $\delta_{\varepsilon_{i1}}^{2}$ = 1). Under a small sample size with γ = 0.29, all the three methods showed extremely poor powers.

**Table 3:** Powers and type I error rates for evaluation of moderation effect.

	$\delta_{\varepsilon_{i1}}^{2}$	Cor	Power (%) ($\gamma_{11}$ ≠ 0)			Type I error (%) ($\gamma_{11}$ = 0)		
			Bs.M	MLR.M	NML.R	Bs.M	MLR.M	NML.R
γ = 0.29 (*N* = 500)	1	0	89.2	92.4	91.6	2.0	1.8	1.0
	1	0.50	91.4	93.8	93.8	2.4	2.2	2.0
	0.50	0	94.0	96.4	96.6	2.0	2.0	2.8
	0.50	0.50	94.6	96.8	97.0	2.8	4.2	3.8
	0	0	98.0	99.8	99.5	2.6	4.4	4.5
	0	0.50	99.0	99.8	100.0	3.8	**7.8**	**5.8**
γ = 0.29 (*N* = 100)	1	0	**18.0**	**28.6**	**27.3**	1.8	4.0	3.2
	1	0.50	**21.3**	**33.4**	**31.3**	2.4	**5.8**	**6.0**
	0.50	0	**25.9**	**38.6**	**35.0**	3.4	**7.2**	**6.3**
	0.50	0.50	**28.8**	**43.6**	**39.9**	2.5	**5.8**	4.5
	0	0	**32.7**	**46.6**	**45.3**	3.4	**11.8**	**5.3**
	0	0.50	**38.2**	**52.8**	**49.5**	3.9	**12.0**	**7.8**
γ = 0.59 (*N* = 500)	1	0	98.4	100	100.0	3.4	3.4	3.4
	1	0.50	98.8	100	100.0	1.8	1.8	1.4
	0.50	0	99.2	100	100.0	3.2	3.8	3.4
	0.50	0.50	98.2	100	100.0	1.8	3.6	3.0
	0	0	99.0	99.8	100.0	5.0	**7**.0	**7.3**
	0	0.50	98.2	100	100.0	4.0	**6.2**	**6.5**
γ = 0.59 (*N* = 100)	1	0	**73.5**	83.8	83.9	2.4	**6.0**	**5.8**
	1	0.50	**78.0**	86.4	87.6	2.6	3.8	4.4
	0.50	0	82.7	87.8	88.1	2.6	**5.2**	4.1
	0.50	0.50	87.9	94.8	94.5	3.6	**6.4**	**6.3**
	0	0	92.3	92.2	95.3	1.0	**10.6**	3.0
	0	0.50	97.4	94.8	98.9	3.3	**10.6**	**6.1**

γ represents the true value of fixed coefficients γ00,γ01, and γ10. Cor represents the true value of the correlation coefficient between variables *X* and *Y*. Values of power lower than 80% and of type I error higher than 5% are indicated by bold type.

For type І error, the Bs.M avoided error inflation effectively and performed best among the three methods. The Bs.M showed relatively stable type І error rates under different conditions. By contrast, when the sample size or true value of $\delta_{\varepsilon_{i1}}^{2}$ decreased, the type І error rates of the MLR.M and NML.R significantly increased and were even larger than 0.05. The correlation coefficient between *X* and *Z* had no significant influence on these results.

Table 4 showed the coverages of the moderation effect under the conditions $\gamma_{11}$ ≠ 0. Under the condition $\gamma_{11}$ = 0, the coverage was exactly 100% minus the type І errors, indicating that the coverage was larger when the type І error was smaller. In Table 4, the Bs.M showed higher coverage (ranging from 95.0% to 98.2%) than the MLR.M and NML.R (ranging from 91.9 to 97.4%) in almost all the conditions. The MLR.M and NML.R performed quite similarly to each other, except for conditions with a small sample size and $\delta_{\varepsilon_{i1}}^{2}$ = 0. Under these conditions, the MLR.M exhibited the lowest coverages of the three methods.

**Table 4:** Coverages of the moderation effect under conditions $\gamma_{11}$ ≠ 0.

	$\delta_{\varepsilon_{i1}}^{2}$	Cor	Bs.M	MLR.M	NML.R
γ = 0.29 (*N* = 500)	1	0	96.0	96.4	99.0
	1	0.50	97.6	97.2	98.0
	0.50	0	97.2	96.4	97.2
	0.50	0.50	97.0	95.0	96.2
	0	0	95.4	93.4	95.5
	0	0.50	96.6	94.8	94.2
γ = 0.29 (*N* = 100)	1	0	96.6	95.8	96.8
	1	0.50	97.2	95.2	94.0
	0.50	0	96.9	93.6	93.7
	0.50	0.50	97.8	94.6	95.5
	0	0	97.6	90.6	94.7
	0	0.50	98.2	90.2	92.2
γ = 0.59 (*N* = 500)	1	0	97.8	97.0	96.6
	1	0.50	97.4	97.0	98.6
	0.50	0	96.4	96.8	96.6
	0.50	0.50	96.8	95.6	97.0
	0	0	95.0	93.6	92.7
	0	0.50	97.8	95.4	93.5
γ = 0.59 (*N* = 100)	1	0	96.0	94.4	94.2
	1	0.50	97.8	95.8	95.6
	0.50	0	96.3	93.2	95.9
	0.50	0.50	98.0	94.4	93.7
	0	0	96.2	87.6	97.0
	0	0.50	97.1	89.6	93.9

γ represents the true value of fixed coefficients $\gamma_{00}$, $\gamma_{01}$, and $\gamma_{10}$. Cor represents the true value of the correlation coefficient between variables *X* and *Y*.

### Estimation accuracy of *R*-square

Tables 5, 6 showed the accuracy of the estimated *R*-squares for the three methods under conditions $\gamma_{11}$ ≠ 0 and $\gamma_{11}$ = 0, respectively. All three methods performed better under the conditions in which $\gamma_{11}$ = 0 (see Table 6) than under those in which $\gamma_{11}$ ≠ 0 (see Table 5). Overall, the Bs.M performed best for the estimation of *R*-square among the three methods, except for the conditions where $\gamma_{11}$ ≠ 0 and $\delta_{\varepsilon_{i1}}^{2}$ = 0. Under the condition $\delta_{\varepsilon_{i1}}^{2}$ = 0, the specified model was equivalent to a traditional MMRM, where the *R*-square is meaningless. All the three method had higher accuracy under the conditions with larger sample size. And as the increase of the true value of $\delta_{\varepsilon_{i1}}^{2}$, the accuracy for the estimation of *R*-square was higher.

**Table 5:** Accuracy of *R*-square for the RMM under the condition $\gamma_{11}$ ≠ 0.

	$\delta_{\varepsilon_{i1}}^{2}$	Cor	Averages of |bias|			MSEs		
			Bs.M	MLR.M	NML.R	Bs.M	MLR.M	NML.R
γ = 0.29 (*N* = 500)	1	0	0.032	0.034	0.035	0.002	0.002	0.002
	1	0.50	0.032	0.035	0.035	0.002	0.002	0.002
	0.50	0	0.055	0.064	0.064	0.005	0.008	0.007
	0.50	0.50	0.056	0.064	0.065	0.005	0.007	0.008
	0	0	0.558	0.378	0.432	0.338	0.174	0.237
	0	0.50	0.568	0.377	0.430	0.344	0.169	0.227
γ = 0.29 (*N* = 100)	1	0	0.061	0.106	0.095	0.007	0.029	0.021
	1	0.50	0.064	0.114	0.090	0.009	0.039	0.018
	0.50	0	0.107	0.210	0.152	0.019	0.091	0.047
	0.50	0.50	0.097	0.188	0.142	0.014	0.072	0.043
	0	0	0.770	0.440	0.659	0.624	0.277	0.486
	0	0.50	0.769	0.438	0.603	0.620	0.273	0.441
γ = 0.59 (*N* = 500)	1	0	0.055	0.057	0.057	0.005	0.005	0.005
	1	0.50	0.054	0.056	0.057	0.005	0.005	0.005
	0.50	0	0.078	0.089	0.089	0.010	0.013	0.014
	0.50	0.50	0.080	0.088	0.089	0.010	0.014	0.014
	0	0	0.262	0.143	0.175	0.077	0.028	0.045
	0	0.50	0.259	0.139	0.169	0.076	0.027	0.042
γ = 0.59 (*N* = 100)	1	0	0.112	0.152	0.139	0.018	0.043	0.035
	1	0.50	0.113	0.153	0.129	0.018	0.047	0.031
	0.50	0	0.173	0.218	0.177	0.041	0.073	0.046
	0.50	0.50	0.158	0.211	0.170	0.034	0.070	0.046
	0	0	0.492	0.191	0.334	0.277	0.072	0.163
	0	0.50	0.483	0.187	0.344	0.263	0.067	0.161

γ represents the true value of fixed coefficients $\gamma_{00},\gamma_{01},\gamma_{10},\mathrm{and}\;\gamma_{11}$. Cor represents the true value of the correlation coefficient between variables *X* and *Y*. True represents the true value of the *R*-square.

**Table 6:** Accuracy of *R*-square for the RMM under the condition $\gamma_{11}$ = 0.

	$\delta_{\varepsilon_{i1}}^{2}$	Cor	Averages of |bias|			MSEs		
			Bs.M	MLR.M	NML.R	Bs.M	MLR.M	NML.R
γ = 0.29 (*N* = 500)	1	0	0.006	0.006	0.006	0.000	0.000	0.000
	1	0.50	0.005	0.005	0.005	0.000	0.000	0.000
	0.50	0	0.011	0.012	0.013	0.000	0.001	0.001
	0.50	0.50	0.010	0.012	0.012	0.000	0.001	0.001
	0	0	0.034	0.085	0.098	0.003	0.023	0.032
	0	0.50	0.035	0.078	0.090	0.004	0.021	0.038
γ = 0.29 (*N* = 100)	1	0	0.027	0.057	0.045	0.003	0.018	0.009
	1	0.50	0.026	0.051	0.046	0.002	0.012	0.009
	0.50	0	0.043	0.107	0.079	0.005	0.038	0.024
	0.50	0.50	0.036	0.094	0.074	0.004	0.032	0.022
	0	0	0.081	0.262	0.146	0.016	0.135	0.060
	0	0.50	0.068	0.251	0.145	0.012	0.126	0.060
γ = 0.59 (*N* = 500)	1	0	0.006	0.007	0.007	0.000	0.000	0.000
	1	0.50	0.005	0.005	0.005	0.000	0.000	0.000
	0.50	0	0.012	0.015	0.015	0.000	0.001	0.001
	0.50	0.50	0.009	0.010	0.010	0.000	0.000	0.000
	0	0	0.038	0.087	0.104	0.004	0.022	0.035
	0	0.50	0.033	0.082	0.088	0.004	0.024	0.029
γ = 0.59 (*N* = 100)	1	0	0.029	0.060	0.054	0.003	0.015	0.014
	1	0.50	0.026	0.055	0.044	0.002	0.015	0.008
	0.50	0	0.041	0.109	0.091	0.005	0.037	0.030
	0.50	0.50	0.038	0.104	0.078	0.005	0.038	0.026
	0	0	0.064	0.239	0.129	0.010	0.115	0.053
	0	0.50	0.064	0.231	0.129	0.012	0.115	0.055

γ represents the true value of fixed coefficients $\gamma_{00},\gamma_{01},\gamma_{10},\mathrm{and}\;\gamma_{11}$. Cor represents the true value of the correlation coefficient between variables *X* and *Y*. True represents the true value of the *R*-square.

## Discussion and conclusion

In this article, the Bayesian approach was investigated to estimate the RMM. We conducted the Bayesian estimation in Mplus and applied the default priors, which are far more common and practicable for empirical researchers. Through a simulation study, the Bayesian approach was compared with the NML.R estimation using the *NML.R* package, as well as the default ML estimation in Mplus. Overall, the Bayesian estimation investigated in this study shows stability in controlling the type I error and outperforms the ML estimations in terms of the accuracy of the effect size *R*-square, as well as the coverage and type I error of the moderation effect. The two ML estimations performed similarly to each other in most conditions except for estimation of the effect size *R*-square, where the NML.R showed extremely poor accuracy.

First, the Bayesian approach had more advantages than the MLR.M and NML.R due to the estimation of the moderation effect. Specifically, the Bayesian method showed higher coverages of the true value of the moderation effect than the other two methods. Moreover, the Bayesian approach had approximately equivalent powers to the MLR.M and NML.R for the estimation of the moderation effect, and its type І error rates were quite well controlled. By contrast, the MLR.M and NML.R had increasingly inflated type І error rates as the sample size and true value of $\delta_{\varepsilon_{i1}}^{2}$ decreased. These results confirmed that the ML estimation was more sensitive to sample size and the magnitude of the random effect of moderation than the Bayesian method. This is reasonable because ML estimation depends on large-sample approximation due to its asymptotic property (Le Cam, 1990; Millar, 2011; Yuan et al., 2014). It is well known that the Bayesian method is appealing for studies with small sample sizes (e.g., Gelman et al., 2003; Yuan and MacKinnon, 2009; Wang and Preacher, 2015). The present study also demonstrated that the Bayesian method was highly efficient even when the random error of the moderation effect was actually zero in the RMM. Nevertheless, if researchers specially pursue high power for estimation of the moderation effect, the two ML estimations remain the qualified methods and should have priority over the Bs.M.

Secondly, in addition to the moderation coefficient, *R*-square plays a crucial role in the RMM as an effect size to quantify the proportion of the given moderator accounting for the random effect in regression. Under the conditions where $\delta_{\varepsilon_{i1}}^{2}$ is nonzero, the Bayesian approach showed highest accuracy among the three methods, and the NML.R performed better than the MLR.M. While Under the conditions where $\delta_{\varepsilon_{i1}}^{2}$ is zero, the RMM is exactly equivalent to the MMRM, which assumes the random effect in regression can be completely explained by the moderator and, thus, *R*-square is meaningless. It is worth noting that the *NML.R* package probably provide a negative value for the estimation of error variance ${\hat{\delta }}_{\varepsilon_{i1}}^{2}$, which would seriously hamper the estimation of the *R*-square.

Thirdly, both the MLR.M and NML.R showed high accuracy for estimation of the regression coefficients, which was consistent with the Bayesian approach. Further, in most conditions, the two ML estimations performed quite similarly to each other in terms of the power, type I error, and coverage for estimation of the moderation effect. However, the NML.R had poor convergence rates, and provided negative value for the estimation of ${\hat{\delta }}_{\varepsilon_{i1}}^{2}$ in most cases. Asparouhov and Muthén (2021) found that ML estimation had a convergence problem for the estimation of the two-level model with latent interactions. The authors considered the reason to be an increase in the numerical integration dimensions in the two-level model possibly exceeding what is computationally feasible (Marsh et al., 2004; Asparouhov and Muthén, 2021). However, the MLR.M is this study converged normally in all conditions. The present study differed from that of Asparouhov and Muthén (2021) in that the model investigated in their study is latent-centered and involves real two-level analysis, where the between-level effect is meaningful. In the current study, one reason for the convergence problem of the NML.R might be that the convergence criterion used in *NML.R* package is strict, in which convergence is considered to be achieved when the maximum update value of all parameters is less than 0.0001 before 300 iterations.

### Limitations

The RMM contributes to moderation analysis with heterogenous residuals, which should violate the assumption of normality and homogeneity of error variance. This study provides a practical and efficient method for estimation of the RMM, making the RMM and its estimation much easier to be implemented. Based on the posterior distribution of the Bayesian estimation, researchers are also allowed to make inferences beyond statistical significance. Nevertheless, this study has limitations. First, only default noninformative priors in the Bayesian estimation were investigated in order to increase the generality of the findings. Alternatively, further research can be conducted to discuss the influence of different prior information, such as different hyper-parameters in the prior distribution (e.g., Lee, 2007), on the estimation.

Second, the study was conducted under the assumption of normality in RMM. There is limited evidence showing how the Bayesian estimation would perform when the normal assumption is violated. Therefore, a further study should be conducted to evaluate Bayesian estimation under the condition that normality is violated and compare it with robust ML estimation.

Third, the performances of the estimation methods are discussed mainly from the perspective of the estimation of moderation effect and effect size. The estimation accuracy with the random effects are also presented in the appendix of this paper. Nevertheless, in applications, researchers may also be concerned with the capability of the estimation method in evaluating the goodness of model fit and selecting between models. This remains to be explored.

Fourth, only the continuous moderator was considered in this study, and theoretically, the RMM is also applicable to the categorical moderator (e.g., Dahlke and Sackett, 2018). Bayesian estimation and ML estimation can be further compared in the context of moderation analysis with the categorical moderator.

Fifth, as in all simulations, data generation in this study could not vary all possible factors. Only the most concerned population parameters were manipulated at several common levels. However, the findings should be representative of what is most common in practice. For further concerns, more complex models, such as those containing several independent variables with multiple interactions or moderated mediation effects, and even a clustered data structure with meaningful between-level effects, could be developed to meet the needs of data analysis in complicated situations.

## Data availability statement

The original contributions presented in the study are included in the article/Supplementary material, further inquiries can be directed to the corresponding author.

## Author contributions

DW contributed to the conception, design, and analysis of data as well as paper drafting and revising of the manuscript. PZ contributed to the critically revising of the manuscript. All authors contributed to the article and approved the submitted version.

## Conflict of interest

The authors declare that the research was conducted in the absence of any commercial or financial relationships that could be construed as a potential conflict of interest.

## Publisher’s note

All claims expressed in this article are solely those of the authors and do not necessarily represent those of their affiliated organizations, or those of the publisher, the editors and the reviewers. Any product that may be evaluated in this article, or claim that may be made by its manufacturer, is not guaranteed or endorsed by the publisher.

## Footnotes

^1^The R package of NML.R can be downloaded at http://www3.nd.edu/~kyuan/moderation/NML.R.

^2^Noting that, in Mplus, there are three types of ML estimations for TYPE = TWOLEVEL RANDOM option, including ML, MLR, and MLF (maximum likelihood parameter estimates with standard errors approximated by first-order derivatives and a conventional chi-square test statistic). The MLR was used by default and denoted as the MLR.M in this study. In this study, consistent results were found for the MLR and the other two ML estimations (i.e., ML and MLF), because the simulated data were normally distributed and fully compliant with the model assumptions.
