# Supplementary material for: Bayesian estimation for the random moderation model: effect size, coverage, power of test, and type І error
Source: Front Psychol. 2023 Jul 3;14:1048842. doi: 10.3389/fpsyg.2023.1048842 (PMC10350495; doi:10.3389/fpsyg.2023.1048842)
Supplement: Supplementary file 1 [file Presentation_1.pdf]

Appendix A

The code for the model definition of the Bs.M.

```
DATA: FILE IS data.dat;
VARIABLE: NAMES ARE X Z Y class;
            USEVARIABLES = X Z Y XZ;
            WITHIN = Y X Z XZ;
            CLUSTER = class;
DEFINE: XZ = X*Z;
ANALYSIS: TYPE = TWOLEVEL RANDOM;
            ESTIMATOR IS BAYES;
            CHAINS = 3;
            PROCESSORS = 2;
            BITERATIONS = (10000);
MODEL:  %WITHIN%
          s | Y ON X;
          Y ON Z;
          Y ON XZ;
          %BETWEEN%
          s;
```

Note:

The ANALYSIS command is used to describe the technical details of the analysis. The TYPE option is used to describe the type of analysis, where TWOLEVEL RANDOM indicates this is multilevel regression model with a random intercept and a random slope varying across clusters. And the ESTIMATOR option identifies the BAYES estimator is selected. CHAINS = 3 indicates three independent Markov chain Monte Carlo (MCMC) chains will be drawn. PROCESSORS=2 will speed up computations. The BITERATIONS option specifies that a minimum of 10,000 and a maximum of the default of 50,000 iterations will be used for each MCMC.

The MODEL command is used to describe the model to be estimated. For TYPE = TWOLEVEL RANDOM, %WITHIN% is used to provide the population parameter values for the individual-level model parameters. The pipe symbol, |, defines a random effect of the slope of Y on X. %BETWEEN% is used to provide the population parameter values for the cluster-level model parameters.

For more details about explanation of each symbols, please see Muthén & Muthén, 1998–2012.

Appendix B

The code for the model definition of the MLR.M.

```
DATA: FILE IS data.dat;
VARIABLE: NAMES ARE X Z Y class;
          USEVARIABLES = X Z Y XZ;
          WITHIN = Y X Z XZ;
          CLUSTER = class;
DEFINE: XZ = X*Z;
ANALYSIS: TYPE = TWOLEVEL RANDOM;
MODEL:  %WITHIN%
        s | Y ON X;
        Y ON Z;
        Y ON XZ;
        %BETWEEN%
        s;
```

Appendix C

The accuracy of error variances in the simulation study.

Table I Averages of |bias| and MSEs of error variances $\hat{\delta}^2_{\varepsilon_{iy}}$ $(\varepsilon_{iy} = e_{iy} + \varepsilon_{i0})$ under the condition $\gamma_{11} \neq 0$.

			Averages of |bias|			MSEs		
	$\delta^2_{\varepsilon_{i1}}$	Cor	Bs.M	MLR.M	NML.R	Bs.M	MLR.M	NML.R
	1	0	0.119	0.113	0.114	0.023	0.02	0.021
	1	0.5	0.123	0.118	0.119	0.024	0.022	0.023
γ=0.29	0.5	0	0.102	0.099	0.099	0.016	0.015	0.015
(*N*=500)	0.5	0.5	0.100	0.099	0.100	0.015	0.015	0.016
	0	0	0.076	0.061	0.088	0.009	0.006	0.012
	0	0.5	0.080	0.063	0.089	0.009	0.006	0.012
	1	0	0.237	0.268	0.273	0.088	0.114	0.119
	1	0.5	0.233	0.257	0.263	0.086	0.102	0.105
γ=0.29	0.5	0	0.194	0.204	0.214	0.061	0.066	0.075
(*N*=100)	0.5	0.5	0.195	0.208	0.215	0.063	0.069	0.080
	0	0	0.156	0.125	0.260	0.045	0.033	0.095
	0	0.5	0.157	0.124	0.220	0.046	0.031	0.075
	1	0	0.123	0.114	0.116	0.024	0.021	0.021
	1	0.5	0.119	0.114	0.115	0.021	0.02	0.020
γ=0.59	0.5	0	0.105	0.103	0.105	0.018	0.017	0.018
(*N*=500)	0.5	0.5	0.111	0.109	0.110	0.019	0.018	0.018
	0	0	0.081	0.064	0.089	0.009	0.006	0.012
	0	0.5	0.081	0.063	0.085	0.009	0.006	0.011
	1	0	0.236	0.258	0.261	0.086	0.101	0.104
	1	0.5	0.242	0.272	0.273	0.095	0.117	0.120
γ=0.59	0.5	0	0.205	0.213	0.228	0.064	0.069	0.079
(*N*=100)	0.5	0.5	0.210	0.224	0.235	0.074	0.082	0.092
	0	0	0.172	0.135	0.252	0.056	0.039	0.099
	0	0.5	0.165	0.135	0.253	0.050	0.037	0.092

Note, γ represents the true value of fixed coefficients $\gamma_{00}, \gamma_{01}, \gamma_{10}$ and γ_{11}. *Cor* represents the true value of the correlation coefficient between variables *X* and *Y*.

Table II Averages of |bias| and MSEs of error variances $\hat{\delta}^2_{\varepsilon_{iy}}$ $(\varepsilon_{iy} = e_{iy} + \varepsilon_{i0})$ under the condition $\gamma_{11} = 0$.

			Averages of |bias|			MSEs		
	$\delta^2_{\varepsilon_{i1}}$	Cor	Bs.M	MLR.M	NML.R	Bs.M	MLR.M	NML.R
	1	0	0.118	0.113	0.113	0.023	0.02	0.021
	1	0.5	0.118	0.112	0.114	0.023	0.02	0.021
γ=0.29	0.5	0	0.107	0.104	0.105	0.017	0.017	0.017
(*N*=500)	0.5	0.5	0.098	0.097	0.099	0.015	0.015	0.015
	0	0	0.080	0.061	0.084	0.009	0.006	0.011
	0	0.5	0.078	0.062	0.082	0.008	0.006	0.010
	1	0	0.241	0.270	0.277	0.090	0.112	0.118
	1	0.5	0.239	0.262	0.266	0.095	0.109	0.111
γ=0.29	0.5	0	0.209	0.228	0.231	0.072	0.085	0.090
(*N*=100)	0.5	0.5	0.204	0.213	0.235	0.067	0.073	0.086
	0	0	0.161	0.132	0.243	0.049	0.035	0.085
	0	0.5	0.161	0.135	0.254	0.051	0.039	0.097
	1	0	0.109	0.104	0.105	0.019	0.017	0.018
	1	0.5	0.119	0.109	0.111	0.022	0.019	0.019
γ=0.59	0.5	0	0.104	0.102	0.103	0.017	0.016	0.017
(*N*=500)	0.5	0.5	0.108	0.102	0.101	0.017	0.016	0.015
	0	0	0.078	0.062	0.084	0.009	0.006	0.011
	0	0.5	0.078	0.060	0.081	0.009	0.006	0.011
	1	0	0.228	0.256	0.250	0.081	0.103	0.100
	1	0.5	0.238	0.259	0.262	0.094	0.113	0.113
γ=0.59	0.5	0	0.201	0.222	0.231	0.067	0.079	0.088
(*N*=100)	0.5	0.5	0.204	0.214	0.230	0.069	0.074	0.088
	0	0	0.158	0.127	0.257	0.050	0.035	0.097
	0	0.5	0.159	0.128	0.260	0.049	0.034	0.099

Note, γ represents the true value of fixed coefficients $\gamma_{00}, \gamma_{01}, \gamma_{10}$ and γ_{11}. *Cor* represents the true value of the correlation coefficient between variables *X* and *Y*.

Table III Averages of |bias| and MSEs of error variances $\hat{\delta}^2_{\varepsilon_{i1}}$ under the condition $\gamma_{11} \neq 0$.

	$\delta^2_{\varepsilon_{i1}}$	Cor	Averages of |bias|			MSEs		
			Bs.M	MLR.M	NML.R	Bs.M	MLR.M	NML.R
	1	0	0.198	0.184	0.184	0.061	0.053	0.053
	1	0.5	0.193	0.174	0.175	0.058	0.049	0.049
γ=0.29	0.5	0	0.138	0.134	0.133	0.030	0.027	0.027
(*N*=500)	0.5	0.5	0.137	0.132	0.134	0.031	0.027	0.028
	0	0	0.120	0.058	0.080	0.018	0.005	0.011
	0	0.5	0.124	0.060	0.082	0.019	0.006	0.011
	1	0	0.523	0.422	0.414	0.441	0.278	0.271
	1	0.5	0.498	0.417	0.411	0.400	0.259	0.252
γ=0.29	0.5	0	0.413	0.304	0.281	0.284	0.135	0.128
(*N*=100)	0.5	0.5	0.412	0.289	0.278	0.272	0.12	0.123
	0	0	0.352	0.093	0.233	0.171	0.026	0.084
	0	0.5	0.345	0.093	0.195	0.170	0.026	0.073
	1	0	0.191	0.176	0.177	0.056	0.049	0.050
	1	0.5	0.190	0.175	0.177	0.058	0.049	0.051
γ=0.59	0.5	0	0.139	0.137	0.138	0.031	0.029	0.030
(*N*=500)	0.5	0.5	0.147	0.137	0.138	0.034	0.031	0.031
	0	0	0.126	0.060	0.081	0.019	0.006	0.011
	0	0.5	0.126	0.060	0.078	0.019	0.006	0.010
	1	0	0.531	0.419	0.418	0.477	0.273	0.274
	1	0.5	0.566	0.431	0.413	0.512	0.301	0.292
γ=0.59	0.5	0	0.436	0.296	0.287	0.307	0.133	0.137
(*N*=100)	0.5	0.5	0.446	0.309	0.295	0.327	0.15	0.151
	0	0	0.376	0.104	0.228	0.201	0.033	0.096
	0	0.5	0.352	0.102	0.228	0.173	0.031	0.084

Note, γ represents the true value of fixed coefficients $\gamma_{00}, \gamma_{01}, \gamma_{10}$ and γ_{11}. *Cor* represents the true value of the correlation coefficient between variables *X* and *Y*.

Table IV Averages of |bias| and MSEs of error variances $\hat{\delta}^2_{\varepsilon_{i1}}$ under the condition $\gamma_{11} = 0$.

	$\delta^2_{\varepsilon_{i1}}$	Cor	Averages of |bias|			MSEs		
			Bs.M	MLR.M	NML.R	Bs.M	MLR.M	NML.R
	1	0	0.190	0.178	0.180	0.058	0.048	0.050
	1	0.5	0.193	0.178	0.181	0.057	0.049	0.050
γ=0.29	0.5	0	0.150	0.144	0.146	0.034	0.031	0.031
(*N*=500)	0.5	0.5	0.135	0.131	0.134	0.028	0.026	0.028
	0	0	0.125	0.058	0.077	0.019	0.005	0.010
	0	0.5	0.124	0.058	0.075	0.018	0.005	0.009
	1	0	0.559	0.448	0.443	0.521	0.305	0.302
	1	0.5	0.511	0.418	0.404	0.419	0.26	0.248
γ=0.29	0.5	0	0.471	0.306	0.293	0.347	0.143	0.147
(*N*=100)	0.5	0.5	0.427	0.293	0.288	0.297	0.132	0.140
	0	0	0.357	0.099	0.216	0.178	0.028	0.076
	0	0.5	0.343	0.102	0.228	0.172	0.033	0.091
	1	0	0.186	0.173	0.175	0.053	0.047	0.048
	1	0.5	0.184	0.174	0.176	0.053	0.047	0.048
γ=0.59	0.5	0	0.144	0.140	0.140	0.034	0.031	0.031
(*N*=500)	0.5	0.5	0.140	0.134	0.134	0.030	0.027	0.027
	0	0	0.124	0.058	0.076	0.019	0.005	0.009
	0	0.5	0.123	0.056	0.074	0.018	0.005	0.010
	1	0	0.511	0.417	0.399	0.419	0.262	0.245
	1	0.5	0.537	0.430	0.426	0.473	0.293	0.284
γ=0.59	0.5	0	0.456	0.311	0.304	0.339	0.14	0.147
(*N*=100)	0.5	0.5	0.403	0.297	0.278	0.277	0.133	0.132
	0	0	0.361	0.098	0.238	0.186	0.029	0.096
	0	0.5	0.341	0.095	0.238	0.165	0.027	0.095

Note, γ represents the true value of fixed coefficients $\gamma_{00}, \gamma_{01}, \gamma_{10}$ and γ_{11}. *Cor* represents the true value of the correlation coefficient between variables *X* and *Y*.
